# Myocardial infarction classification and its implications on measures of cardiovascular outcomes, quality, and racial/ethnic disparities

**DOI:** 10.1002/clc.23431

**Published:** 2020-08-11

**Authors:** Aaron L. Hilliard, David E. Winchester, Tanya D. Russell, Rosland D. Hilliard

**Affiliations:** ^1^ College of Pharmacy and Pharmaceutical Sciences Florida Agricultural and Mechanical University Tallahassee Florida USA; ^2^ Division of Cardiovascular Medicine, Department of Medicine, College of Medicine University of Florida Gainesville Florida USA; ^3^ Cardiology Section, Medical Service Malcom Randall VA Medical Center Gainesville Florida USA; ^4^ Center for Advanced Professional Excellence University of Colorado Anschutz Medical Campus Aurora Colorado USA; ^5^ Health Matters Environmental, Medical, Pharmaceutical and Toxicology Jacksonville Florida USA

**Keywords:** cardiac, diagnostic codes, international statistical classification of diseases ICD 10, myocardial injury, myocardial infarction < ischemic heart disease, plan‐do‐check‐adjust, quality improvement, troponin

## Abstract

Heart disease continues to be the leading cause of death in the United States, with approximately 805 000 cumulative deaths from myocardial infarctions (MI) from 2005 to 2014. Gender and racial/ethnic disparities in MI diagnoses are becoming more evident in quality review audits. Although recent changes in diagnostic codes provided an improved framework, clinically distinguishing types of MI remains a challenge. MI misdiagnoses and health disparities contribute to adverse outcomes in cardiac medicine. We conducted a literature review of relevant biomedical sources related to the classification of MI and disparities in cardiovascular care and outcomes. From the studies analyzed, African Americans and women have higher rates of mortality from MI, are more probably to be younger and present with other comorbidities and are less probably to receive novel therapies with respect to type of MI. As high‐sensitivity troponin assays are adopted in the United States, implementation should account for how race and sex differences have been demonstrated in the reference range and diagnostic threshold of the newer assays. More research is needed to assess how the complexity of health disparities contributes to adverse cardiovascular outcomes. Creating dedicated medical quality teams (physicians, nurses, clinical documentation improvement specialists, and medical coders) and incorporating a plan‐do‐check‐adjust quality improvement model are strategies that could potentially help better define and diagnose MI, reduce financial burdens due to MI misdiagnoses, reduce cardiovascular‐related health disparities, and ultimately improve and save lives.

## 1 INTRODUCTION

In the United States, heart disease continues to be the leading cause of death in men and women regardless of race or ethnic group.^1^, ^2^, ^3^ With respect to racial/ethnic groups in the United States, collectively African American adults experience a higher rate of cardiovascular diseases, compared to white adults.^4^ This is expected to continue into the future, as it is estimated that 45.1% of the US population will have some form of heart disease by 2035.^5^ Unless the prevalence of heart disease is better controlled and significantly reduced, total direct medical care costs related to heart disease are expected to increase from $318 billion to $749 billion.^5^ Within the spectrum of heart disease, someone in the United States has a myocardial infarction (MI) every 40 seconds.^5^ There have been recent discussions involving the misdiagnosis of MI and its implications on patient care and medical costs. The reclassification of the International Statistical Classification of Diseases and Related Health Problems (ICD) 10 codes in 2017 helped further define the types of MI, yet more work is needed to educate medical professionals on a more useful way of making a bedside clinical diagnosis of MI and prevent medical coding errors. Further, elevated cardiac troponin (cTn) has been associated with disparities in race and gender‐related care.^2^, ^6^ Inconsistencies in clearly distinguishing a type of MI, widening racial and ethnic gaps in health, and gender‐related differences in biomarker analyses contribute to the incongruency of diagnosis and medical coding of MI, leading to unnecessary costs and death. Currently there is not enough data on the nexus of MI misdiagnoses and health disparities, and how both attribute to medical coding and cardiac medicine as a whole. Thus, it is imperative to get a better understanding of these challenges from a quality perspective to improve patient treatments and outcomes. We will highlight some of these challenges and present potential strategies to address these challenges in this review.

## 2 DISTINGUISHING DIFFERENCES IN MI CLASSIFICATION

Despite efforts to improve health among disadvantaged racial and ethnic groups in the United States, health disparities continue to exist. The interactions between the many factors that attribute to these disparities, and how they result in different outcomes, are not always well defined. Thus, before discussing health disparities of racial/ethnic groups with respect to MI one must first dissect the disparities within the classification of MI. The standard clinical definition of MI signifies the presence of an acute myocardial injury detected by abnormal cardiac biomarkers, namely cTn, in the setting of evidence of acute myocardial ischemia.^7^ A precise, definitive diagnosis not only provides valuable guidance on treatment, patient prognosis, and lifestyle changes, but it is also associated with policy and resource allocation, medical coding‐related diagnosis, and hospital reimbursement.^7^, ^8^ Due to the secondary characteristics of multiple cardiac events, it is necessary to further delineate and define the various types of MI for proper diagnosis. Type 1 MI is an MI with clinical evidence of ischemia caused by atherosclerotic plaque disruption resulting in coronary thrombosis and detection of a rise and/or fall of cTn values with at least one value above the 99th percentile upper‐reference limit (URL).^7^, ^8^ In addition, patients must also exhibit one of the following symptoms of myocardial ischemia conditions: new ischemic electrocardiogram changes, development of pathological Q waves, evidence of new loss of viable myocardium or new regional wall motion abnormality in a pattern consistent with ischemic etiology via imaging, and identification of a coronary thrombus by angiography or autopsy.^7^ Type 2 MI also includes the aforementioned symptoms, but is due to a supply‐demand mismatch of myocardial oxygen in the absence of coronary thrombosis.^7^, ^8^ Prior to 2017, differentiating patients with type 1 and type 2 MI‐based via medical coding was not possible because an ICD code for each particular MI subtype did not exist.^8^ Without strict ICD 10 coding criteria and an effective means of determining the specific type of MI at bedside and given that patients with type 2 MI can potentially have numerous underlying comorbidities, a type 2 diagnosis is subject to uncertainty or misdiagnosis. An estimated 90% of type 2 MI patients were not being coded.^8^, ^9^ An ICD code now exists for type 2 MI, yet issues still surround accurately documenting other forms of type 1 MI as type 2 MI. Another potential misclassification exists between myocardial injury and MI.

### 2.1 Myocardial injury vs MI

Myocardial injury is defined as elevated cTn values, at least one value above the 99th percentile URL, and is considered acute if there is a rise and/or fall of cTn values.^7^ MI is a subset within acute myocardial injury. In this review, the use of “injury” throughout the manuscript is intended as injury without MI. The most current universal definition of MI states the importance of the differentiation of MI from myocardial injury and further differentiation from other types of MI, especially type 2 MI.^7^ The definition of acute myocardial injury states that it is characterized by a rise and/or fall of cTn, the most agreed upon standard being >20% change for patients with normal baseline cTn or a > 50% change for patients with a baseline elevated Tn; however there is currently still discrepancy on exactly how much cTn is needed in order to distinguish the injury as acute. A number of patients with elevated cTn have no clinical evidence of ischemia (ie, no ischemic chest symptoms and no ECG changes). Without ischemia, a diagnosis of type 2 MI should not be made.^9^ Yet, in a single‐center observational study, McCarthy et al found that approximately 42% of type 2 MI patients were misdiagnosed and actually had myocardial injury without ischemia.^10^ Currently, there is no ICD 10 code for myocardial Injury. A specific ICD 10 code for myocardial injury would reduce misdiagnosis and improve the characterization of a patients' condition. Myocardial injury is also a recent focus of many quality improvement and value‐based programs.

Recent emphasis on quality improvement efforts adhering to guidelines and ensuring proper MI diagnosis have been connected to performance metrics and reimbursement. One such effort, the Hospital Readmission Program (HRRP), was designed to improve the quality of patient post‐acute care and reduce Medicare spending by preventing rehospitalizations of conditions such as acute MI.^8^ Acute MI is one of six conditions included in the HRRP, with a combined total of $528 million being withheld from hospitals due to readmissions in 2017.^8^ Of all readmitted patients with MI contributing to the HRRP penalty, about 10% may have actually had type 2 MI.^8^, ^11^ In conjunction with the Hospital Value‐Based Purchasing Program, and after strict adjudication with physician medical record reviewers using the most current universal definition of MI,^7^ McCarthy et al found that approximately 41% of patients with nonischemic myocardial injury are frequently misdiagnosed and subsequently billed as having type 2 MI.^10^ Compared to patients with myocardial injury, patients type 2 MI also exhibited a higher prevalence of cardiovascular‐related comorbidities such as coronary artery disease (50.6% vs 33.2%; *P* < .001), heart failure (52.4% vs 37.4%; *P* < .001), peripheral arterial disease (23% vs 12.1%; *P* < .001) and prior MI(21.8%vs 14.3%; *P* = .02).^10^ A limitation to this study is the single‐center observational analysis. Further multicenter analyses and development of evidenced‐based guidelines and diagnostic coding strategies to manage the various types of MI and myocardial injury are warranted to fully understand the implications of myocardial injury and type 2 MI misdiagnoses on quality improvement efforts and overall patient health.

### 2.2 STEMI, NSTEMI, and type 2 MI

Type 1 MI can be further divided into two classifications: ST‐elevation myocardial infarction (STEMI) and non‐ST‐elevation myocardial infarction (NSTEMI). STEMI is defined as an acute coronary thrombosis or persistent ST‐segment elevation of ≥1 mm in ≥2 contiguous electrocardiographic leads.^7^, ^12^ NSTEMI is defined as ischemic symptoms at rest from an acute coronary plaque rupture or erosion, lasting ≥10 minutes, occurring within 24 hours before hospital admission, and displaying either elevated cardiac biomarkers (either creatine kinase or cTn) within 24 hours after initial presentation.^12^ Prior to the introduction of the ICD‐10 code for type 2 MI, patients with type 2 MI were often coded as a NSTEMI or with no code at all.^9^, ^13^ While STEMI care is well defined and fairly consistent in delivery, NSTEMI management is substantially more variable on the patient, clinician, facility, and regional levels..^4^ Since there is now a specific code for type 2 MI, and type 2 MI does not feature acute coronary thrombotic plaque disruption, great efforts should be made by clinicians to not identify it as NSTEMI.^13^ Doing such could have adverse effects on patient prognosis, treatment, and outcome. Improved methods of distinguishing type 1 and 2 MI could further enhance the study of MI and the potential development of MI‐specific treatments.

## 3 DISPARITIES IN MI OUTCOMES AND CLASSIFICATION

Tackling health disparities can be daunting given that the work involves not only patient‐doctor interactions, but also how this interaction involves patient personal preferences, trust level, education, and socioeconomic factors; unfortunately, these interactions are often not well characterized.^3^ In addition, many physicians may have little to no training on how to incorporate or even recognize health disparities. Some physicians may also shift the sole responsibility of addressing health disparities to the society as a whole or the government. Despite the ongoing efforts of government entities such as the Institute of Medicine and the National Institute of Minority Health, African Americans still carry the highest burden of heart disease and mortality rates compared to white Americans.^3^, ^4^, ^14^ African Americans also have higher mortality rates from MI.^15^ Further, cardio‐related comorbidities such as hypertension, insulin resistance, diabetes mellitus, dyslipidemia, obesity, and chronic kidney disease are more prevalent in African Americans.^3^ Since a few of these comorbidities coincide with features of type 2 MI, recent studies have begun to examine disparities in various types of MI among African Americans. To our knowledge, there are not yet studies specifically assessing the interplay of specifically type 1 and type 2 MI and health disparities. Nonetheless, two recent studies have shed light on disparities in STEMI and NSTEMI.

Both studies highlight not only MI disparities in African Americans collectively, but also gender differences. Heart disease is the number one killer of women worldwide regardless of race or class.^2^, ^16^ Both biological and social factors contribute to the gender disparities seen in heart disease, as there are differences in the pathobiology of acute MI which lead to misdiagnosis.^2^ This appears to also be the case for African Americans as a whole, especially as it pertains to the higher prevalence of comorbidities in this population which both increase the incidence of MI and the adverse effects after MI.^17^ TRIUMPH (Translational Research Investigating Underlying disparities in acute MI Patients' Health status), a multicenter, prospective study, is on the forefront of disparity research by obtaining detailed sociodemographic, clinical, treatment, health status, metabolic, and genetic information from patients recovering from MI and assessing of their outcomes.^17^ Creating and accessing more databases and registries such as TRIUMPH that include all patients with various types of MI could help clarify the differences in metabolic and genetic characteristics in MI outcomes, and potentially help eliminate cardiovascular health disparities.

### 3.1 Disparities by STEMI and NSTEMI

Race has previously been implicated with disparate management of NSTEMI, with African American patients being less likely to receive novel, expensive therapies or undergo more invasive procedures compared to whites.^3^, ^18^, ^19^, ^20^, ^21^ Anstey et al examined differences in care related to sex and race in patients presenting with STEMI or NSTEMI using the ACTION Registry‐GWTG database, a national quality improvement database.^12^ African American patients were more likely to present with STEMI or NSTEMI to an academic hospital covered by Medicare, Medicaid, or self‐insured compared to white patients who were most likely covered by an health maintenance organization or private insurance.^12^ NSTEMI was found to be more prevalent than STEMI in African American patients.^12^ African American patients were also younger yet had a higher incidence of comorbidities such as hypertension, diabetes mellitus, and stroke.^12^ Interestingly gender differences were also evident, as African American women had a higher frequency of diabetes mellitus compared to their counterparts.^12^

While acute medical treatment was similar among African American and white women, African American men had significantly lower overall rates of being prescribed an anticoagulant.^12^ Table 1 displays additional disparities seen in African American patients presenting with STEMI and NSTEMI after adjustment for baseline comorbidities and socioeconomic status (SES).^12^

**TABLE 1:** STEMI and NSTEMI racial disparities (adapted from Anstey et al^12^)

African American women presenting with STEMI (compared to White women)	African American men presenting with STEMI (compared to White men)
Similar rates of overall reperfusion	Similar rates of overall reperfusion
Similar rates of diagnostic catheterization	Similar rates of catheterization
Similar primary percutaneous coronary intervention (PCI) for STEMI	Similar primary PCI for STEMI
Significantly lower rates of coronary artery bypass grafting (CABG) and revascularization	Significantly lower rates of CABG and revascularization

African American women presenting with NSTEMI (compared to White women)	African American men presenting with NSTEMI (compared to White men)
Less likely to have interventional procedures and early invasive therapy	Significantly less likely to undergo catheterization
Longer median time to percutaneous coronary intervention (PCI)	Longer median time to percutaneous coronary intervention (PCI) and less likely to undergo PCI within 48 h
Less likely to undergo revascularization and coronary artery bypass grafting (CABG)	Less likely to undergo revascularization and coronary artery bypass grafting (CABG)

Abbreviations: NSTEMI, non‐ST‐elevation myocardial infarction; STEMI, ST‐elevation myocardial infarction.

In the ARIC (Atherosclerosis Risk in Communities) Community Surveillance study, Arora et al analyzed hospital surveillance of NSTEMI in four US communities over a 15‐year period.^4^ Similar to the ACTION Registry‐GWTG database study, African American patients were less insured, younger, and exhibited a higher incidence of comorbidities compared to their white counterparts.^4^ African American patients were also less likely to receive the following evidence‐based NSTEMI therapies compared to white patients: aspirin (85% vs 92%), nonaspirin antiplatelet therapy (45% vs 60%), β‐blockers (85% vs 88%), and lipid‐lowering medications (68% vs 76%).^4^ Such as the ACTION Registry‐GWTG database study, African American patients hospitalized with NSTEMI were significantly less likely to undergo invasive angiography (45% vs 61%) or revascularization (25% vs 45%) compared to white patients even when risk‐adjusted.^4^ An additional feature of this study analyzed comorbidities and clinical course, demonstrating that African American patients had lower probabilities of receiving nonaspirin antiplatelet therapy, lipid‐lowering agents, aspirin, and undergoing angiography and revascularization.^4^

An important feature of health disparity research is the inclusion of social determinants of health and/or demographics into patient medical charting to help health care professionals give quality care and treatment.^22^ SES has been recently used to assess outcome for patients with STEMI undergoing reperfusion. In 2014, Agarwal et al analyzed 372 984 patients with a principal diagnosis of STEMI as defined by ICD‐9 codes over a 9 year period from 2003 to 2011.^23^ SES was assessed by median household incomes of patients classified into quartiles: quartile 1 = $1 to $37 999; quartile 2 = $38 000 to $47 999, quartile 3 = $48 000 to $62 999, and quartile 4 = $63 000 or more. Inhospital mortality was higher among patients in the lower quartile compared to the highest quartile (odds ratio [95% CI]: 1.11 [1.06 to 1.17]), and there was a significant trend toward reduced timely reperfusion among patients in the lower quartiles than those in the higher quartiles.^23^ Although the incidence of inhospital mortality among patients who had delayed or no reperfusion was higher than those who underwent timely reperfusion (*P* < .001), there was no significant difference in the inhospital mortality incidence across the SES quartiles.^23^ However, patients in the lower quartiles who underwent timely reperfusion had a significantly higher incidence of inhospital mortality compared to those in the higher quartiles.^23^ Women also were found to have had higher inhospital mortality rates compared to men across all SES quartiles (*P* < .001).^23^ While this study found a higher proportion of nonwhite in the lower socioeconomic quartiles, it does not necessary delineate racial or ethnic disparities seen across various SES strata. However, it gives credence to the importance of including such data into medical charting to give context to a better understanding of patient health outcomes.

### 3.2 Implications of cTn levels on disparities

The use of cardiac‐specific biomarkers is not only critical for defining, providing specific diagnosis and/or prognosis, and ensuring accurate decision‐making in the management of acute MI, but also provides information about associated pathways and/or consequences of MI, which may help to further understand population specific characteristics.^24^, ^25^ The discovery of cTn in 1963 created a paradigm shift in the ability to use a more sensitive cardiac‐specific biomarker to optimize a diagnosis of MI.^7^ However, using the reigning gold standard is not without caveats. Elevated cTn is not only specific for myocardial injury but also for the other various subtypes of MI and other comorbidities. As mentioned previously, heart disease is the leading cause of death in women. Along with biological and social factors being key factors in gender‐related disparities of heart disease, Shah et al have also hypothesized that differences in the reference range and diagnostic threshold of cardiac biomarkers such as cTn between women and men is also a viable factor.^2^ While older generation cTn had been generally thought as a good gender‐independent biomarker, studies in the 1990s by Hamm et al showed that cTn T levels measurable in only 27% of women compared to 43% of men (2, 31). Wiviott et al found that men were more likely to have elevated cTn I, cTn T, and creatine kinase‐MB compared to women when older generation cTn assays with a single diagnostic threshold were used (2, 32). Even newer, high‐sensitivity markers have revealed that the 99th percentile reference limits are up to 2‐fold higher in men (2, 33). This has sparked an argument for the development of gender‐specific thresholds of troponin given that cTn levels are consistently lower in women than men, spanning ethnic populations; cTn levels in stable populations suggest different prognostic information with respect to gender, with stronger associations noticed in women even when uniform thresholds are utilized; gender‐specific thresholds for cTn I show women are at high risk of future MI and death; and women are less likely to receive evidence‐based therapy and have worse outcomes in overall acute coronary diseases.^2^ A gender‐specific cTn diagnostic criteria may help to address these disparities, as well as provide valuable information in the development of new cardiac biomarkers.

Disparities in cTn could also have an impact with respect to cardiovascular‐related services. While the current definition of MI encourages judicious use of cTn in testing, misdiagnoses could potentially increase the amount of resources needed to correctly diagnose the MI leading to nontrivial risks costs.^6^ Thus, we recently hypothesized that patients with elevated cTn would undergo more testing than those without elevated cTn. Using a retrospective study cohort of 26 663 subjects, 18.6% had at least one elevated cTn assay while acute MI was diagnosed in 3.9%.^6^ We found that men were more likely to undergo catheterization and cardiology consultation compared to women (OR 1.29, 95% CI 1.20 to 1.39 and OR 1.45, 95% CI 1.31 to 1.61).^6^ In addition, African American patients were less likely to have either catheterization (OR 0.85, 95% CI 0.77 to 0.93) or consultation (OR 0.72, 95% CI 0.63 to 0.82) performed.^6^ Although limited by our inability to derive a statistical method to determine the change is cTn (ie, a rise and/or fall) to accurately distinguish the type of MI, our data implies that an indiscriminate use of cTn may be incongruous with best clinical practice. It also stresses the continuous need to focus efforts on strategies to include social determinants of in the further development and adoption of high‐sensitivity cTn assays.

## 4 DISCUSSION

Accurate coding of MI is invaluable for ultimately improving patient prognosis and outcomes as well as assessing value‐based programs and hospital quality metrics.^13^, ^26^ A concerted effort between physicians, nurses, medical coders, and other members of cardiovascular care teams is key to ensuring correct ICD 10 coding of MI occurs and, thus, overall quality improvement (QI). A physician may have a working diagnosis upon admission; however, on discharge that diagnosis may no longer be active. Patients with elevated cTn may present with MI, but the specific type and treatment is not elaborated upon in the medical chart. Cardiovascular care teams may not know or understand how inclusion of SES and other pertinent social determinants of health in medical charts could potentially impact health outcomes. Inefficient closed‐loop communication among hospitalists, specialists, and medical coders on MI diagnoses can cause costly critical inpatient care. These scenarios create conundrums for cardiovascular medical teams who need more information on how patients are treated and for what type of MI. An inability to distinguish the evidence‐based treatments for type 1 MI from treatments that are not evidence‐based and are dependent on a secondary diagnosis, as is the case with type 2 MI, could be harmful and potentially fatal. Equally important, miscoding type 2 MI as a myocardial injury on initial diagnosis could have substantial negative financial outcomes as such data would be captured under readmission penalties and/or value‐based programs. McCarthy et al found that 30‐day readmission rates for nonischemic myocardial injury were higher than the national 30‐day readmission rates for acute MI (21% vs 14.8%), and that many patients with type 2 MI were included in Hospital Readmission Reduction Program penalties; hospitals that include patients with myocardial injury misclassified as having type 2 MI may perform more poorly in value‐based payment programs.^10^ Thus, accurate diagnostic coding is also crucial to prevent potential costly penalties.

A potential bedside diagnosis strategy to improve the coding conundrum among cardiovascular medical teams is the use of a clinical documentation improvement specialist (CDI). Improved documentation could not only benefit hospital reimbursement by ensuring patients are not over or under diagnosed, but also influence overall quality and safety measures by improving communication strategies among medical teams. Using trained nurses as CDIs, Swaminath et al conducted a pilot study to determine if (1) real‐time bedside communication can improve physician response; (2) increasing accuracy of documentation based on CPT billing codes could increase billing accuracy; and (3) real time, efficient communication among physicians and CDIs improved the severity of illness and mortality risk.^27^ The pilot model allowed for real‐time communication by incorporating a CDI on rounds with residents and attending physicians compared to a control model of an attending physician requesting supplemental information (ie, query response) from a CDI post hoc.^27^ Query response rate, severity of illness, and mortality risk were measured in a cardiac intensive care unit over a 3‐month period with three cardiology physicians who implemented the pilot model; performances were compared to their prior year's performance within the same 3‐month time frame).^27^ Despite a lower volume of cases during the real‐time communication pilot period, query response rate increased during the pilot period compared to the control period (63 responses vs 40 responses).^27^ The accuracy of severity of illness and mortality risk also improved during the pilot period (1 expired patient with low ROI and mortality risk) compared to the control period (3 expired patients with low ROI and mortality risk code).^27^ Although this was a small study and did not directly assess MI, this study shows the potential benefit of including and consistently using CDIs as part of a cardiovascular medical team to accurately diagnose and document patients with MI. It also had an advantage of analyzing real‐time feedback between physicians and CDIs at a teaching facility where residents are more likely to complete documentation. Implementing strategies such as including social determinants of health and real‐time communication with CDIs give residents an opportunity to learn about the importance of documentation and to implement these practices early in their careers.

A strategic and collaborative approach is needed to ensure additional documentation from a hospitalist, attending physician, and/or cardiovascular specialist is included in the patient chart to assist in the identification of myocardial injury and MI type 1 or 2 for medical coders and billers. New guidelines have been presented but are not actively and consistently used to code MI type 2 during clinical visits or hospitalizations. A clear plan is still needed to ensure that a type 1 MI is not coded as a type 2 MI, or a myocardial injury as any other type of MI. In addition, the higher prevalence of comorbidities in African American patients with MI calls for a sharper focus on patient medical history combined with additional social determinants of health including, but not limited to, mental health, substance addiction and abuse, homelessness, and access to resources. While effective educational materials that address all of these issues should be created and adapted, physicians, clinical residents, medical coders, nurses, and CDIs should also meet as a team to review documentation in clinical cases. This could lead to more accurate coding of the diagnostic codes carried out on the patient. Development of documentation, coding, and social determinants of health training protocols, along with routine updating and the ability to make changes made in real‐time, could be beneficial to overall QI. Thus, a systematic approach with practical solutions is needed to ensure QI in cardiovascular MI diagnosis and documentation.

**FIGURE 1:**
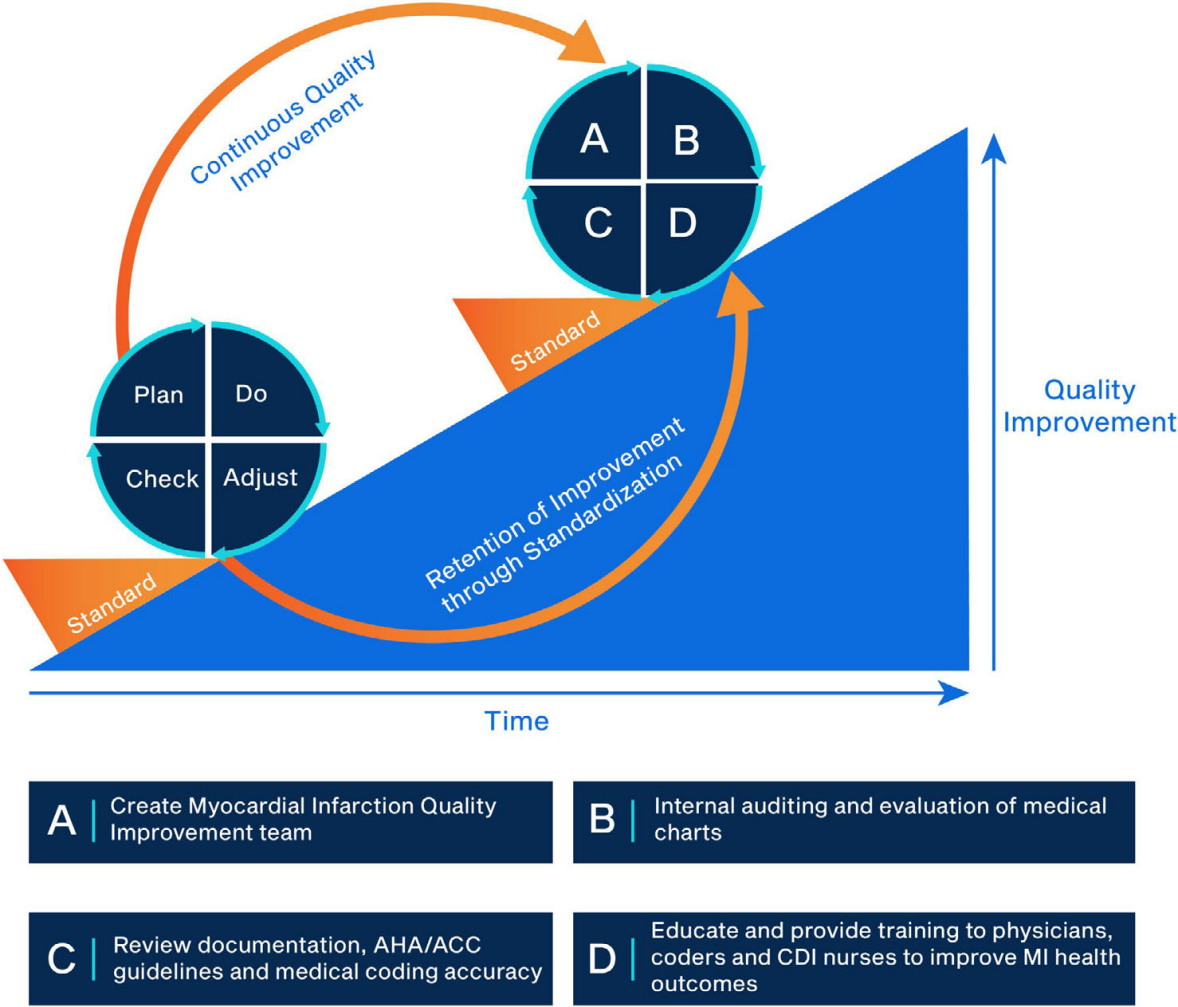
PDCA approach to sustain MI quality improvement (adapted from^29^). CDI, clinical documentation improvement specialist; MI, myocardial infarctions; PDCA, plan‐do‐check‐adjust

Taking the Swaminath et al study further, an “MI quality improvement (MIQI) team” comprised of a physician, resident, CDI nurse, medical coder, and QI specialist could potentially have a substantial impact on MI diagnosis and documentation. With adequate institutional support, a QI approach could be used by an MIQI team for real time, bedside communication. An example is a QI model used by the Fellows' Applied Quality Training program at the University of Florida to give residents hands‐on experiential training in QI.^28^ Two key aspects of using this approach among these fellow were (1) the highest yield QI projects were those that made a substantial impact for minimal effort and (2) persistence in making necessary changes, studying the outcome, and implementing or adjusting as needed.^28^ Figure 1 depicts a similar repetitive and continuous QI approach, a plan‐do‐check‐adjust (PDCA) cycle,^29^ that may be applicable for an MIQI team. In the P (plan) phase, the MIQI team could be created as well as protocols to learn about and stay current on updated coding and documentation practices. The D (do) phase could involve internal auditing and evaluation of charts of challenging MI cases, taking into account information on MI diagnosis, race, gender, and social determinants of health. A crucial part of this cycle is the C (check) phase, where real‐time discussion on documentation and coding accuracy according to AHA/ACC guidelines could be addressed; recognition of shortcomings could also be included in this phase as a check on QI. Finally, the A (adjust) phase could allow corrections to be made in an appropriate time frame (within two to 3 days of discharge if not at bedside). This phase could also be used to educate and provide training to physicians, coders and CDI nurses to help identify key areas of shortcomings as needed. By consistently using a PDCA cycle, the retention of improvement through standardization could increase over time, leading to a potential decrease of institutional financial burdens due to MI misdiagnosis, over or underuse of cardiovascular services and penalties of coding. Continuous QI practices could also increase awareness of health disparities and its impact on MI diagnosis and treatment; and, ultimately increase overall patient outcomes and health.

## 5 CONCLUSION

The evolving and expanding epidemic of comorbidities such as obesity, hypertension, metabolic syndrome and diabetes disproportionately affects minorities and women, and these metabolic disturbances both increase the incidence of type 2 MI and the adverse outcomes after an MI.^12^, ^17^ Steps have been taken to explore the role of race and gender disparities in MI and biomarker thresholds on the proper identification and diagnosis of various MI subtypes and offer strategies to implement more efficient bedside feedback and documentation among cardiovascular medical teams. Creating dedicated interdisciplinary medical quality teams and incorporating a PDCA QI model are strategies that could potentially help reduce cardiovascular‐related health disparities and ultimately improve and save lives.

## CONFLICT OF INTEREST

The authors declare no conflict of interest.

## AUTHOR CONTRIBUTIONS

The authors contributed equally to the conceptualization, writing, review, and editing of the manuscript.
